# Analyzing the effect of physical activity on cyberattack behavior in college students using the chain mediation model

**DOI:** 10.3389/fpsyg.2025.1653542

**Published:** 2025-10-22

**Authors:** Hongbo Zhao, Bo Chen

**Affiliations:** School of Physical Education, Liaoning Normal University, Dalian, China

**Keywords:** physical activity, cyberattack behavior, moral excuses, rumination thinking, college students

## Abstract

**Introduction:**

Cyberattack behavior is an undesirable phenomenon among college students in China that cannot be ignored, and it has always been under the close scrutiny of researchers. This study investigated and analyzed 536 college students using the Physical Activity Rating Scale, the Cyber Aggressive Behavior Scale, the Moral Excuse Scale, and the Rumination Thinking Scale. The aim was to explore the mediating effect of moral excuses and rumination thinking on the relationship between physical activity and cyberattack behavior in college students.

**Findings:**

(1) Physical activity, moral excuses, rumination thinking, and cyberattack behavior among college students are correlated in pairs. (2) Physical activity negatively predicted moral excuses and cyberattack behavior as well as rumination thinking. Moral excuses positively predicted rumination thinking and cyberattack behavior while rumination thinking positively predicted cyberattack behavior.

**Conclusion:**

Moral excuses and rumination thinking both individually and chain-mediate the relationship between physical activity and cyberattack behavior among college students.

## Introduction

In the information age, the internet has become an integral part of people’s lives. According to the 55th Statistical Report on the Development of the Internet in China, the number of internet users in China reached 1.108 billion as of December 2024, with an internet penetration rate of 78.6 percent (China Internet Network Information Center, 2025). Digital products and services are becoming increasingly integrated into people’s daily lives, affecting how they learn, work, and communicate in various scenarios, such as instant messaging, online payments, online shopping, searching for news and advice, and entertainment and gaming. However, while they provide convenience, their anonymity, openness, and accessibility have led to undesirable behaviors during network use, especially among college students. Cyberattack behavior is particularly prevalent in this group (Liang et al., 2023). A substantial body of research has indicated that a minimum of 49.7% of college students have perpetrated two or more acts of aggression against a particular individual or group via the Internet (Tanrikulu and Erdur-Baker, 2021). Individuals who have been exposed to cyberattacks may experience a range of adverse emotional consequences, including anxiety, anger, depression, and impaired social functioning. These effects may manifest as attention deficits, interpersonal tensions, and suicidality (Zhou, 2023; Bonanno and Hymel, 2013; Litwiller and Brausch, 2013; Pabian and Vandebosch, 2016). Therefore, there is an urgent need to take effective measures to improve the situation. The cathartic theory posits that physical activity can function as a positive and effective intervention, playing a role in the alleviation of stress and anxiety by increasing the brain’s arousal level, thereby enhancing an individual’s mental well-being (Wang, 2024), it is also effective in improving individual cyberattack behavior (Li, 2024). However, the mechanism through which physical activity influences college students’ cyberattack behavior requires further study. Consequently, this study innovatively constructs a chain mediation model of “physical activity-moral excuses-rumination thinking-cyberattack behavior” with the aim of conducting a profound analysis of the internal mechanism of physical activity on college students’ cyberattack behavior. This study provides a new theoretical basis and practical direction for colleges and universities to carry out cyberattack behavior education and intervention.

### The relationship between physical activity and cyberattack behavior of college students

The advent of the third technological revolution has given rise to a novel form of aggressive behavior known as cyberattack behavior, which is also referred to as cyberbullying. This emerging phenomenon can be considered a derivative of traditional attack behavior (Zhou, 2023). Wong-Lo et al. (2011) posit that the behavior of cyberattacks is closely related to the development and popularization of the Internet. They further argue that cyberattacks do not exist independently, but rather as an extension of traditional attack behavior, characterized by more complex and covert features. Physical activity is a type of sport that promotes fitness, beauty, recreation, leisure, healthcare, rehabilitation, and psychological and intellectual exercise. The goal is to enhance physical fitness, improve physical and mental health, and maintain the body’s abilities (Xi, 2004). Social control theory posits that the maintenance of social order hinges on the strength of bonds between individuals and society. Stronger bonds correlate with fewer deviant behaviors, while weaker bonds increase the likelihood of such actions. Physical activity can strengthen social bonds by increasing participation in social activities, reinforcing collective normative beliefs, and fostering emotional attachment. Combined with its promotion of endorphin and dopamine secretion to alleviate negative emotions, it collectively restrains individual behavior and reduces cyberattack behavior (Ross, 1989).

A body of research has indicated that physical activity can function as a preventative measure or a mitigating factor in regard to cyberattack behavior exhibited by college students (Li, 2024). Regular physical activity has been demonstrated to influence cognitive, emotional, and behavioral processes, potentially leading to a shift in the experience of these domains (Jiang et al., 2017), participation in physical activity has been shown to facilitate university students’ ability to maintain composure and concentration in the face of adversity, thereby enhancing their capacity to manage stress and frustration (Qi, 2024), it is imperative to mitigate the probability that individuals will engage in cyberattack behavior as a means of emotional release (Wang, 2023). Therefore, this study proposes Hypothesis 1 (H1): physical activity has a negative relationship with cyberattack behavior among college students.

### Mediating effects of moral excuses

Moral excuses are defined as specific cognitive dispositions that individuals develop in order to minimize the perceived harm of their actions. These cognitive dispositions include redefining actions to reduce perceived responsibility for the consequences of actions and reducing identification with the pain of the injured target (Bandura, 1986a; Bandura, 2017; Bandura, 1999; Bandura, 2002). Moral excuses are defined as cognitive mechanisms that facilitate the acceptance of unethical behavior, exerting a substantial influence on individual conduct (Bandura et al., 1996). A growing body of research has demonstrated that sports participation exerts a positive influence on the cultivation of college students’ awareness of rules. This heightened awareness is accompanied by a notable enhancement in external attribution and excuse-making skills. Furthermore, sports participation fosters the development of enhanced self-discipline and a more profound comprehension of the significance of rules during physical activity. These effects culminate in a substantial reduction in the prevalence of moral excuses, thereby guiding college students toward more ethical conduct (Qu, 2021). Self-regulation theory posits that individuals achieve goals and adapt to environments by monitoring gaps between themselves and their objectives and adjusting their actions accordingly. This process relies on self-regulatory resources. When resources are abundant, individuals can anchor moral goals, monitor cognitive biases, reduce tendencies to weaken moral constraints, and lower acceptance of cyberattacks. When resources are scarce, individuals experience diminished focus on moral goals and weakened regulatory capacity, making them more likely to justify cyberattacks and increasing the likelihood of cyberattack behavior occurring (Baumeister et al., 1998). A substantial body of research has identified a tendency among individuals to employ cognitive restructuring strategies as a means of reducing their sense of moral constraints on cyberattacks. This phenomenon, often referred to as “moral excuses, “has been found to positively predict cyberattack behavior among college students (Miao et al., 2023). In the aftermath of posting online remarks of an injurious nature, some college students endeavor to rationalize their cyberattack behavior by invoking the defense of “just joking, no malice.” They may perceive such behavior as a form of “joke” or “venting, “thereby overlooking the deleterious consequences it inflicts on others. Some individuals may perceive cyberattacks as a form of “joking” or “venting,” leading to a disregard for the harm they inflict on others. The moral excuses mechanism has been demonstrated to have a number of functions, including the ability to mitigate or even eliminate feelings of guilt that arise in individuals when they engage in unethical behaviors, such as cyberattacks. This mitigation can be achieved through the mechanism’s two main properties: punishment avoidance and self-defense (Bandura, 1986b). Consequently, college students who frequently employ moral justifications are more prone to engage in cyberbullying behaviors. Therefore, this study proposes Hypothesis 2 (H2): moral excuses mediate the relationship between physical activity and cyberattack behavior of college students.

### Mediating effects of rumination thinking

According to Nolen-Hoeksema, rumination thinking is a response style in which individuals repeatedly focus on a negative emotional, thought, or behavioral state that is relevant to them and think repeatedly about the causes and consequences of this negative state. However, the individual does not actively think about how to solve the real problem. Consequently, rumination thinking is a form of maladaptive performance (Nolen-Hoeksema, 1991). According to the Mind-flow theory, physical activity can serve as a means of reducing ruminative thinking in college students by inducing a state of mind-flow (Ye et al., 2022), the experience of focus and pleasure in the mindstream has been demonstrated to impede the occurrence of negative thought cycles and enhance emotional regulation. A concomitant effect of this experience is a reduction in ruminative thinking, which, in turn, has been shown to weaken hostile perceptions and aggressive impulses (Jin et al., 2018). Concurrently, the psychological intervention value embedded in physical activity can effectively inhibit the development of negative rumination thinking (Si, 2023). Research has demonstrated that individuals who engage in ruminative thinking exhibit a propensity for cyberattack behavior (Yang et al., 2021). Individuals exposed to violent environments, such as those who have been criticized, blamed, or insulted by others, are susceptible to ruminative thinking. This phenomenon can result in impaired executive functioning, psychophysiological deficits, and the activation of aggression schemas, which can increase the propensity for cyberattack behavior in cyber environments (Si, 2023). Therefore, the present study proposes Hypothesis 3 (H3): rumination thinking mediates the relationship between physical activity and cyberattack behavior among college students.

### Chain mediation effects of moral excuses and rumination thinking

Research has shown a positive correlation between moral excuses and rumination thinking, specifically, individuals with elevated levels of moral excuses frequently seek to rationalize their behavior, attributing their shortcomings to external circumstances or other individuals. This tendency serves to intensify ruminative thinking (Gan, 2023). The cognitive-emotional interaction theory demonstrates that cognition and emotion dynamically influence each other. Cognition shapes the interpretation and experience of emotions, while emotions guide the direction and processing efficiency of cognition. Together, they regulate an individual’s judgments, decisions, and behavioral responses. When an individual exhibits a high degree of moral excuses, they often seek to rationalize their own behavior by shifting blame to external circumstances or others. This tendency intensifies rumination thinking (Wells and Matthews, 1994). The higher the level of moral excuses, the more likely individuals are to be driven by feelings of relative deprivation into rumination thinking, persistently dwelling in negative emotions and cognitions. During this process, their positive attitudes toward aggressive behavior are continually reinforced, gradually forming aggressive tendencies, ultimately leading to a significant increase in cyberbullying (Pan et al., 2021). Therefore, the present study puts forth Hypothesis 4 (H4): moral excuses and rumination thinking play a chain mediating role between physical activity and cyberattack behavior among college students.

**Figure 1:**
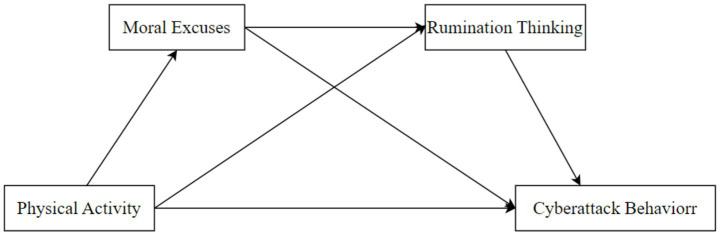
Hypothetical model (theoretical underpinnings for each pathway in the figure are detailed in the concluding remarks of Section 1.4.).

Consequently, the research hypothesis model was formulated as depicted in Figure 1.

The core theoretical underpinnings for each pathway in Figure 1 are as follows: (1) Physical activity → cyberattack behavior; (2) Physical activity → moral excuses → cyberattack behavior; (3) Physical activity → rumination thinking → cyberattack behavior; (4) Physical activity → moral excuses → rumination thinking → cyberattack behavior. Each theory explains the logical interactions between variables along its corresponding pathway, consistent with the model’s assumptions.

## Methods

### Participants

In this study, a sample of 536 college students from various institutions in Liaoning. Province was selected for survey participation through the convenience sampling method. The distribution of the online questionnaire utilized the Questionnaire Star platform, which encompassed five sections: basic information, physical activity level, cyberattack behavior, moral excuses, and rumination thinking. Liaoning Province, a prominent educational center in China, boasts a multitude of institutions of higher learning that admit students from diverse geographical areas throughout China. The present study encompasses a diverse sample of students from various educational institutions, academic disciplines, and academic levels, thereby offering a more comprehensive representation of the characteristics of Chinese college students as a whole. All respondents in this study signed an informed consent form. The following criteria were stipulated for the inclusion of participants: first, they were required to be full-time undergraduate students between the ages of 18 and 25; second, they were required to be free of any physical illness or psychiatric history. In the course of the screening process, 19 questionnaires were excluded due to their regular or incomplete nature. Consequently, 517 valid questionnaires were recovered, yielding an effective recovery rate of 96.46%. Of the participants, 218 (42.2%) were male college students and 299 (57.8%) were female college students. The demographic profile of the survey respondents is delineated in Table 1.

**Table 1:** Basic information on survey respondents (*N* = 517).

Demographic variables	Categorization	Number of people	Percentage (%)
Gender	Men	225	43.52%
	Female	292	56.48%
Grade	First Year	249	48.16%
	Sophomore Year	117	22.63%
	Junior Year	57	11.03%
	Senior Year	94	18.18%

## Research methodology

### Physical activity measurements

The physical activity level of college students was assessed using Liang (1994) revised Physical Activity Rating Scale. The scale consists of three dimensions, each with one question: intensity of exercise (e.g., how intensely do you engage in physical activity?), duration of exercise (e.g., how long do you engage in physical activity at one time?), and frequency of exercise (e.g., how many times do you engage in physical activity per month?). A five-point Likert scale was used, with intensity and frequency scored from 1 to 5 and time scored from 0 to 4. Physical activity level was calculated by multiplying physical activity intensity, frequency, and time, with higher scores indicating greater physical activity. The Cronbach’s alpha of the physical activity rating scale in this study was 0.770. Content validity was good (CVI = 0.89, standard: CVI ≥ 0.80), and structural validity met criteria (single-factor structure, factor loadings 0.68–0.75, CR = 0.77, AVE = 0.53, standard: factor loadings ≥0.50, CR ≥ 0.70, AVE ≥ 0.50).

### Cyberattack behavior measurements

The Cyberattack Behavior Scale, developed by Zhao and Gao (2012), was utilized to assess the cyberattack behavior of college students. The scale under consideration consists of two dimensions: instrumental aggression and reactive aggression. The instrumental aggression subscale comprises 15 questions, such as “I have impersonated another person on the Internet to do something that has caused damage to that person’s reputation.” The reactive aggression subscale consists of 16 questions, including “I have stolen equipment from other gamers in an online game while playing the online game.” The total number of questions is 31. The scale is based on a 4-point scale ranging from 1 (never) to 4 (always). After scoring each dimension individually, the scores for each item of these two dimensions are summed. Higher scores indicate higher levels of cyberattack behavior in an individual. The alpha (Cronbach’s) of the cyberattack behavior scale in this study was 0.870, with an internal consistency coefficient of 0.726 for the instrumental aggression subdimension and 0.868 for the reactive aggression subdimension. Content validity was good (CVI = 0.92, standard: CVI ≥ 0.80), and structural validity met criteria (two-factor structure, factor loadings 0.56–0.82; CFA validation *χ*^2^/df = 2.31, RMSEA = 0.05, CR = 0.73/0.87, AVE = 0.51/0.58; standards: *χ*^2^/df < 3, RMSEA < 0.08, CR ≥ 0.70, AVE ≥ 0.50).

### Moral excuses measurements

Adapted from Bandura (1986c) and Wang and Yang (2010) undertook a revision of the Chinese version of the Moral Excuses Scale, with the objective of assessing the moral excuses of college students. The scale consists of moral defenses (e.g., “making fun of someone does not really hurt them”), euphemistic labels (e.g., “a child who merely instigates another person to break discipline should not be blamed for the fact that the other person listened to the instigation”), favorable comparisons (e.g., “a child should not be chastised for the wrongs committed by the group to which they belong”), and responsibility shifting (e.g., It’s okay to fight to protect your friends), and blame diffusion (“It’s okay to tell a little lie because it does not really hurt anyone”). The scale has eight dimensions, each comprised of four questions, for a total of 32 questions. Damaging something is not as serious as hitting someone, and blame attribution (e.g., shoving or jostling a classmate is just a joke). There are eight dimensions, each comprised of four questions, for a total of 32 questions. There were no reverse scoring questions. A 5-point Likert scale was used, ranging from 1 (completely disagree) to 5 (completely agree). After scoring each dimension individually, the scores for each item in the eight dimensions were summed. Higher scores represent higher levels of moral excuses. The Cronbach’s alpha of the moral excuses scale in this study was 0.965. Content validity was good (CVI = 0.91, standard: CVI ≥ 0.80), and construct validity met criteria (eight-factor structure, factor loadings 0.59–0.84, CR = 0.97, AVE = 0.56, standard: factor loadings ≥0.50, CR ≥ 0.70, AVE ≥ 0.50).

### Rumination thinking measurements

This analysis was prepared using the methods outlined by Nolen-Hoeksema and Morrow (1991), the revised Rumination Thinking Scale by Han and Yang (2009). Rating College Students’ Rumination Thinking. The scale under consideration is composed of three dimensions: Symptom Rumination, Reflective Thinking, and Compulsive Thinking. Symptom Rumination is characterized by recurrent thoughts concerning feelings of loneliness. Reflective Thinking involves introspection and examination of the underlying causes of ongoing ruminations. Compulsive Thinking involves the documentation of thoughts and subsequent analysis. The scale comprises 12 questions pertaining to Symptom Rumination, 5 questions concerning Reflective Thinking, and 5 questions related to Compulsive Thinking, totaling 22 questions. It is noteworthy that the scale does not incorporate reverse scoring questions. The scale is based on a 4-point scale ranging from 1 (never) to 4 (always). After each dimension is scored individually, the scores for each individual item of these four dimensions are summed. The resulting sum is the total score of the Rumination Thinking Scale. Higher scores indicate higher levels of rumination thinking in an individual. In the present study, the alpha (Cronbach’s) of the rumination thinking scale was determined to be 0.966. Content validity was good (CVI = 0.88, standard: CVI ≥ 0.80), and construct validity met criteria (three-factor structure, factor loadings 0.55–0.81, CR = 0.97, AVE = 0.54, standard: factor loadings ≥0.50, CR ≥ 0.70, AVE ≥ 0.50).

### Statistical processing

The data were found to be valid and were, thus, imported into SPSS 27.0 software for analysis. The researchers employed a variety of analytical techniques, including Pearson correlation analysis, regression analysis, and chained mediation analysis. A chained mediation analysis was conducted, employing the bootstrap method of the Process plug-in for mediation effect testing. Model 6 in the SPSS macro program Process was used to test the mediating effects.

## Results

### Common method bias test

Initially, conventional methods were subject to procedural control through the implementation of anonymized measurement protocols, randomization of scale sequence, and other such techniques. The phenomenon of method bias must be considered. Secondly, the Harman one-way test was employed to rule out common method bias resulting from the questionnaire method. The results of the factor analysis demonstrated that there were 15 factors with an eigenroot greater than 1, and the amount of variance explained by the first factor was 24.2%, indicating that there was no obvious common method bias.

### Descriptive statistics and correlation coefficients for each variable

A correlation analysis was conducted to ascertain the relationship between physical activity and college students’ cyberattack behavior, moral excuses, and rumination thinking. The analysis utilized the mean scores of each variable. The findings revealed a significant negative correlation between physical activity and college students’ cyberattack behavior, moral excuses, and rumination thinking. Additionally, a significant positive correlation was identified between moral excuses and cyberattack behavior and rumination thinking. Furthermore, a significant positive correlation was observed between rumination thinking and college students’ cyberattack behavior. The analysis also revealed a significant negative correlation between gender and physical activity. Additionally, a significant positive correlation was identified between grade level and physical activity (Table 2).

**Table 2:** Mean, standard deviation and correlation coefficient of each variable (*n* = 517).

Variant	*M*	SD	1	2	3	4	5	6
Gender (1)	1.60	0.49	1					
Grade (2)	1.94	1.12	0.234^**^	1				
Physical activity (3)	3.05	1.06	−0.389^**^	0.230^**^	1			
Cyberattack behavior (4)	1.50	1.37	−0.153^**^	0.123^**^	−0.229^**^	1		
Moral excuses (5)	1.70	0.90	−0.027	−0.053	−0.186^**^	0.262^**^	1	
Rumination thinking (6)	1.56	0.80	0.008	−0.079	−0.120^**^	0.179^**^	0.552^**^	1

*p < 0.05, **p < 0.01, ***p < 0.001, same below. Gender: 1 = male college students, 2 = female college students; Grade: 1 = freshman, 2 = sophomore, 3 = junior, 4 = senior.

### Analysis of the chain-mediated effects of stress perception and rumination thinking

Significant correlations were found between both physical activity, moral excuses, rumination thinking, and college students’ cyberattack behavior, which meets the statistical requirements for further mediation effect analyses of moral excuses and rumination thinking. This study used the Process macro program in SPSS, controlling for gender and grade level, and chose a Bootstrap sample size of 5,000 at the 95% confidence interval to test the mediating effect of moral excuses and rumination thinking.

The results of the multiple regression analysis are shown in Table 3. Physical activity has a significant negative predictive effect on cyberattack behavior (*β* = −0.55, *p* < 0.001). After including moral excuses and rumination thinking in the regression equations with physical activity, the predictive effect of physical activity on cyberattack behavior remains significant (*β* = −0.12, *p* < 0.001). Physical activity has a significant negative predictive effect on moral excuses (*β* = −0.65, *p* < 0.001) and rumination thinking (*β* = −0.25, *p* < 0.001). The negative predictive effect of physical activity on cyberattack behavior was significant (*β* = −0.65, *p* < 0.001), as was the negative predictive effect on rumination thinking (β = −0.25, *p* < 0.001). The negative predictive effect of physical activity on moral excuses and rumination thinking is significant (*β* = −0.65 and −0.25, respectively, *p* < 0.001). The negative predictive effect of physical activity on cyberattack behavior is significant [(*β* = −0.65, *p* < 0.001) 0.25, *p* < 0.001]. Moral excuses were significant positive predictors of rumination thinking (*β* = 0.79, *p* < 0.001) and cyberattack behavior (*β* = 0.34, *p* < 0.001). Rumination thinking was a significant positive predictor of cyberattack behavior (*β* = 0.27, *p* < 0.001) (Table 3).

**Table 3:** Regression analysis of the relationship of variables in the model.

Regression equation		Overall fit index			Significance of regression coefficients		
Outcome variable	Predictor variable	R	R2	F	β	SE	t
Cyberattack behavior	Genders	0.49	0.24	55.10***	−0.50	0.09	−5.39***
	Grade				0.08	0.04	2.07*
	Physical activity				−0.55	0.04	−12.81***
	Genders				−0.62	0.11	−5.67***
Moral excuses	Grade	050	0.25	57.25***	0.13	0.05	2.89*
	Physical activity				−0.65	0.05	−12.97***
	Genders				0.00	0.06	−0.05
	Grade				0.02	0.02	0.87
Rumination thinking	Physical activity	0.88	0.78	453.77***	−0.25	0.03	−7.96***
	Moral excuses				0.79	0.02	32.33***
	Genders				−0.16	0.07	−2.22*
	Grade				0.00	0.03	0.05
cyberattack behavior	physical activity	0.77	0.59	144.64***	−0.12	0.04	−3.06**
	Moral excuses				0.34	0.05	6.96***
	Rumination thinking				0.27	0.05	5.39***

***p < 0.001; **p < 0.01; *p < 0.05.

**Figure 2:**
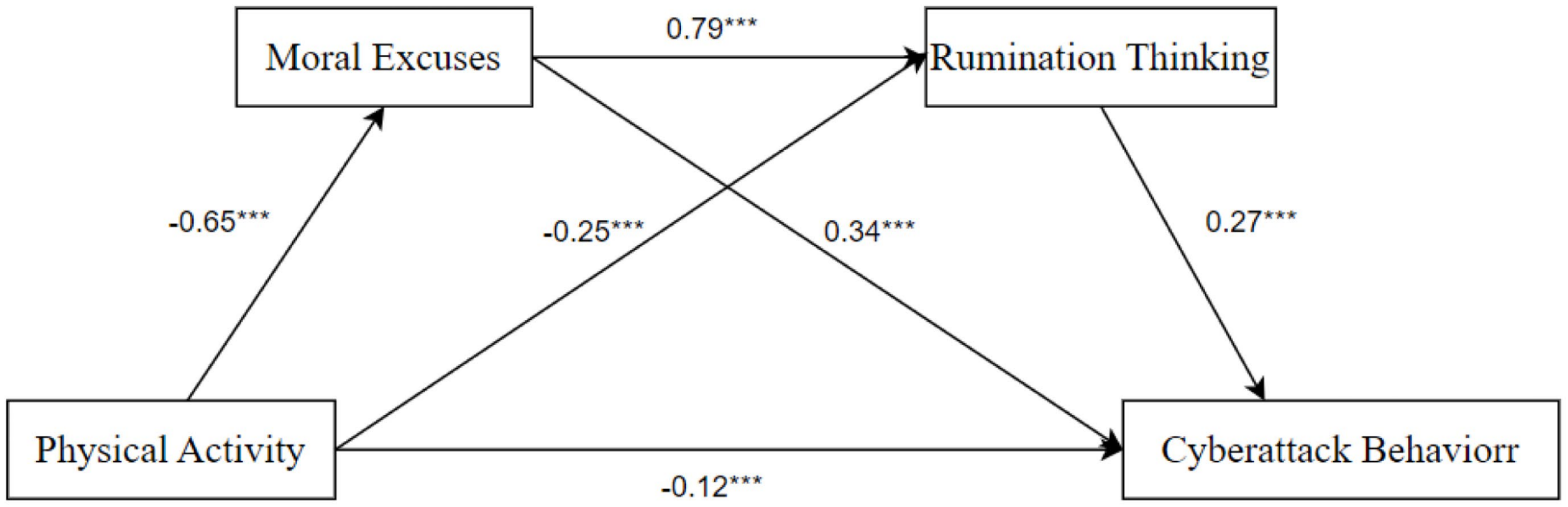
Path of physical activity’s effect on cyberattack behavior. ***p < 0.001, **p < 0.05, **p < 0.01.

Second, the mediating effect test was conducted. Table 4 shows that moral excuses and rumination thinking play a mediating role in the relationship between physical activity and college students’ cyberattack behavior, with a mediating effect value of −0.43. The 95% confidence interval is [−0.58, −0.28], which does not include 0, indicating that the mediating effect is significant, accounting for 78.18% of the total effect of physical activity on college students’ cyberattack behavior (−0.55). The mediating effect includes three indirect effect paths: indirect effect 1 (−0.22) through the pathway of “physical activity – moral excuses – cyberattack behavior”; 95% of the confidence intervals do not contain 0, indicating that the mediating variable has a significant indirect effect, accounting for 78% of the total effect of physical activity on college students’ cyberattack behavior (−0.55). The indirect effect of the mediator variable is significant, accounting for 40.00% of the total effect of physical activity on college students’ cyberattack behavior (−0.32). Indirect effect 2 (−0.07) through the pathway of “physical activity – rumination thinking – cyberattack behavior”; the 95% confidence interval does not contain 0, indicating that the indirect effect of the mediator variable is significant, accounting for 40.00% of the total effect (−0.32) of physical activity on college students’ cyberattack behavior. The total effect (−0.32) is 12.72%. Indirect effect of “physical activity – moral excuses – rumination thinking – cyberattack behavior” 3 (−0.14); 95% confidence interval does not contain 0, indicating that the indirect effect of the mediating variable is significant, accounting for 12.72% of the total effect of physical activity on college students’ cyberattack behavior. The indirect effect of physical activity on cyberattack behavior is 25.45% of the total effect of physical activity on cyberattack behavior of college students (−0.32). A two-by-two comparison of the indirect effects of different paths showed that none of them were significantly different. The detailed path model of physical activity affecting cyberattack behavior is shown in Figure 2.

**Table 4:** Results of bootstrap mediated effects analysis.

Pathway	Efficiency value	Boot standard error	Boot CI lower bound	Boot CI Upper Limit	Relative mediation effect
Aggregate effect	−0.55	0.43	−0.63	−0.46	
Direct effect	−0.18	0.04	−0.19	−0.42	
Total indirect effect	−0.43	0.07	−0.58	−0.28	78.18
Indirect effects 1	−0.22	0.07	−0.35	−0.09	40.00
Indirect effect 2	−0.07	0.02	−0.13	−0.02	12.72
Indirect effects3	−0.14	−0.06	−0.26	−0.04	25.45

## Discussion

This study reveals the chain-mediated role of moral excuses and rumination thinking in the relationship between physical activity and cyberattack behavior among college students. Theoretically, it enriches research on the influence mechanisms of cyberattack behavior. Practically, it provides a solid theoretical foundation for enhancing physical activity among college students. This also offers crucial evidence for universities and educational authorities to develop scientifically sound educational guidance strategies.

### The relationship between physical activity and cyberattack behavior

The study shows that there is a significant negative correlation between physical activity and cyberattack behavior of college students, i.e., the higher the level of physical activity, the lower the incidence of cyberattack behavior, which verifies the hypothesis H1 of this paper, and is consistent with the results of existing studies (Qi, 2024). Lu et al. (2014) demonstrated that physical activity can help college students build social confidence and reduce shyness through low-pressure social settings such as team sports and group fitness activities. This, in turn, makes them more inclined toward rational communication in online interactions, thereby lowering the probability of cyberattack behavior. In his research on the cyberattack behavior of college students, Huang (2019) made an observation that adverse emotions, such as anxiety and depression, are significant triggers of cyberattack behavior. Furthermore, he determined that physical activity can substantially reduce the activation intensity of such emotions through multidimensional neurobiological mechanisms. The following procedures are intended to promote the generation of new neurons in the hippocampus and prefrontal cortex of the brain, increase the density of synaptic connections, and enhance the efficiency of neural signaling. These procedures also aim to optimize the brain from a passive stress state to active regulation by activating the brain-derived neurotrophic factor and other molecular mechanisms. Finally, the promotion of endorphins and dopamine is intended to follow. The activation of brain-derived neurotrophic factors (BDNF) and other molecular mechanisms has been demonstrated to optimize the nucleus accumbens-noradrenergic pathway and the limbic dopaminergic pathway in the midbrain. This optimization enables the brain to shift from a passive stress state to an active regulatory state, thereby promoting the secretion of endorphins and dopamine, which in turn induce a sense of emotional pleasure. Li et al. (2024) further demonstrated that physical activity serves as a healthy conduit for emotional catharsis among college students. In the face of challenges such as academic pressure and interpersonal conflicts, exercise functions as a psychological safety valve, facilitating the release of negative emotions and thereby preventing the escalation of these accumulated psychological energies into cyberattack behaviors. Secondly, positive emotional experiences (e.g., a sense of achievement, a sense of group belonging) generated during exercise have been shown to foster the development of psychological protective factors, thereby further attenuating the generative basis of aggressive tendencies (Lu et al., 2014). Consequently, researchers have acknowledged the notion that physical activity can influence college students’ cyberattack behavior.

### The role of moral excuses in mediating the relationship between physical activity and cyberattack behavior among college students

The findings of the study indicated that physical activity influenced college students’ cyberattack behavior through the mediating role of moral excuses. The results demonstrated a significant negative prediction of moral excuses by physical activity, and the higher the level of moral excuses, the higher the college students’ cyberattack behavior. This validates hypothesis H2 of this paper and aligns with the results of previous studies (Baumeister et al., 1998; Miao et al., 2023). A substantial body of research has demonstrated that students who regularly partake in physical activity exhibit notable benefits in terms of self-discipline, group cohesion, and emotional stability (Yang et al., 2013), and the more self-regulating the individual, the lower the level of moral excuses tends to be (Bandura, 1999). Furthermore, engagement in physical activity has been demonstrated to foster the development of positive attitudes toward life and healthy values. In addition, it has been shown to promote a heightened sense of self-improvement. This shift in mindset has been found to contribute to increased rationality and tolerance, thereby reducing the likelihood of aggressive responses to cyber conflicts (Li, 2024). A comparison of traditional cyberattack behaviors with the anonymity, rapid dissemination (Schneider et al., 2015), and lack of temporal and spatial limitations characteristic of cyberattacks reveals the challenges in subjecting individual behaviors in cyberspace to ethical constraints (Nathanson, 2001). This phenomenon also indirectly contributes to an increase in the activity of college students within the cyber environment. The failure of moral monitoring functions in the virtual cyber environment is more likely to result in cyberattack behavior from individuals who exhibit verbal and aggressive tendencies in the real environment (Miao et al., 2023). Individuals exhibiting higher psychological stress responses demonstrate online deviant behaviors closely linked to impaired moral cognition. They tend to interpret and attribute online stimuli with hostility, exhibit emotional and cognitive extremism, thereby impairing normal moral judgment. This leads to cyberbullying attacks or triggers online public opinion crises (Miao et al., 2023). Therefore, moral excuses serve as a distinct intermediary between physical activity and cyberattack behavior among college students.

### The mediating role of rumination thinking between physical activity on college students’ cyberattack behavior

The present study found that physical activity influenced college students’ cyberattack behavior through the mediating effect of rumination thinking. This validates hypothesis H3 of this paper and is consistent with the results of existing studies (Si, 2023; Dai, 2021). Research has demonstrated that a conducive environment conducive to physical activity promotes the secretion of neurotransmitters in the brain, regulates mood, and reduces stress (Penedo and Dahn, 2005), which in turn reduces rumination thinking (Nolen-Hoeksema et al., 1994). Also, the fitness value of physical activity and the value of psychological interventions can be effective in suppressing rumination thinking (Si, 2023). The emergence of rumination thinking enhances an individual’s perception of adversity-related information while consuming substantial cognitive resources, leading to heightened hostility levels. This results in simplistic and crude problem-solving approaches, ultimately triggering aggressive behavior (Zhao and Zhang, 2023). According to the principles of attack model theory, the behavior of college students in regard to cyberattacks is associated with environmental input variables, including exposure to violence and aggression, among others. Additionally, the individual’s information processing process plays a significant role in this relationship. The exposure of college students to violent environments has been demonstrated to result in the processing of information related to violence as an input variable. This exposure has been shown to lead to persistent thinking about the harm and trauma brought by violent events, resulting in the emergence of rumination thinking. In turn, this rumination thinking has been demonstrated to cause college students to narrow the scope of attention, reduce self-control, and implement cyberattack behavior in accordance with the activated attack schema (DeWall et al., 2011). Based on the theory of cognitive resource appropriation (Peng, 2006). It is an established fact that individuals have limited cognitive resources, and they engage in rumination thinking. As a specific thinking mode, rumination’s generation and persistence will take up a significant amount of individuals’ limited cognitive resources. The over-occupation of cognitive resources by rumination thinking has been demonstrated to result in a reduction in the ability to exercise self-control. This is due to the aforementioned lack of cognitive resources, which in turn activates the potential attack mode within the individual. This, in turn, increases the possibility of aggressive cyber behavior (Jin et al., 2018). Thus, moral excuses play a separate mediating role between physical activity and cyberattack behavior among college students.

### Chain mediation of moral excuses and rumination thinking

The results of this study show that moral excuses and rumination thinking play a chain mediating role between physical activity and college students’ cyberattack behavior, i.e., an increase in the level of moral excuses leads to an increase in rumination thinking, and moral excuses can significantly and positively predict rumination thinking, and there is a positive correlation between the two, which verifies the hypothesis of this paper, H4, and is in line with the results of existing studies (Pan et al., 2024). Studies have shown that physical activity significantly decreases the number of moral excuses made by college students (Miao et al., 2023). Some college students post insulting remarks online and use the excuse “just joking, no malice” to justify their cyberbullying behavior. They may regard cyberbullying as a kind of “joke” or “venting” and ignore the harm it causes. They may view cyberattacks as a form of “joking” or “venting” and disregard the harm they cause. Individuals can reduce or even eliminate the guilt they feel when engaging in unethical behaviors (e.g., cyberattacks) by avoiding punishment and using moral excuses to defend themselves (Bandura, 1986b). The integration of college students into the realm of physical activity has been demonstrated to yield a multitude of benefits, including the cultivation of a heightened sense of self-restraint. This heightened sense of self-restraint, in turn, has been shown to foster a propensity toward problem-solving, a tendency to assume personal responsibility for one’s actions, and a reduction in the tendency to attribute external causes or blame others for personal shortcomings. Consequently, this heightened sense of self-restraint serves to mitigate the presence of moral justifications (Zhang et al., 2020). Individuals with low moral excuses maintain normal moral self-regulation functions. Their internal feelings of guilt and responsibility regarding behavioral consequences form effective constraints. Even when negative events trigger rumination thinking, they can suppress aggressive behavior through internal scrutiny of moral norms (Hou et al., 2024). Conversely, when individuals have high levels of moral excuses, rumination thinking has a stronger predictive effect on aggression. This makes it easier for individuals to rationalize their behavior and more likely that they will commit aggressive acts (Zhang et al., 2020), in particular, cyberattack behavior. To summarize, the present study posits that moral excuses and rumination thinking play a mediating role in the relationship between physical activity and college students’ cyberattack behavior.

### Limitations and future prospects

This study explores the relationship between physical activity and college students’ cyberattack behavior. From a theoretical standpoint, this study contributes to the extant research on the relationship between physical activity and college students’ cyberattack behavior. It elucidates the mediating role of moral excuses and rumination thinking in this context and offers an original perspective on the factors that influence college students’ cyberattack behavior. In practice, the results of this study offer practical guidance directions for preventing and intervening in college students’ cyberattack behavior. It is imperative for educational institutions and broader society to recognize the pivotal role of physical activity in fostering positive behaviors among college students. To this end, the formulation of policies that encourage active participation in physical activities and the implementation of diverse physical activities are crucial. These measures are designed to enhance the level of physical activity among college students, thereby promoting their well-being and academic success. However, it is important to note that the study is not without its limitations. Firstly, the study employed a questionnaire survey method, and the data were based on college students’ self-reports, which may have introduced some subjective bias. Future research endeavors may benefit from the incorporation of objective measurements, such as the utilization of cyberattack behavior monitoring software, to systematically document college students’ cyberattack behavior. Secondly, the present study employs a cross-sectional research design. Subsequent studies may consider adopting a longitudinal or experimental research design to further verify the causal relationship between physical activity, moral excuses, rumination thinking, and cyberattack behavior. This study is limited in its scope, as it exclusively explores the mediating role of moral excuses and rumination thinking. Further research is necessary to elucidate the relationship between physical activity and college students’ cyberattack behavior, as well as to identify other potential influencing variables, such as personality traits and self-control. Consequently, subsequent studies will encompass multivariate variables and methodically investigate the interaction mechanism between variables, with the objective of elucidating the path of physical activity’s influence on college students’ cyberattack behavior with greater precision.

## Conclusion

This study investigates the impact of physical activity on college students’ cyberattack behavior and the mediating mechanisms of moral excuses and rumination thinking. Findings reveal that moral excuses and rumination thinking exert both independent and joint chain-mediated effects between physical activity and cyberattack behavior, influencing cyberattack behavior through three indirect pathways. This breaks through the limitations of previous studies that often examined single mediating variables in isolation. It innovatively constructs and validates the chained mediation model of “physical activity—moral excuses—rumination thinking—cyberattack behavior,” revealing for the first time the chained transmission mechanism of moral excuses and rumination thinking in this relationship. This provides a more systematic explanation of the underlying logic through which physical activity influences college students’ cyberattack behavior, deepening understanding in this field. Furthermore, the findings provide scientific grounds for universities and educational authorities to develop prevention and intervention strategies against cyberattacks among college students. Specifically, enhancing students’ physical exercise levels can curb cyberattacks by reducing moral excuses and rumination thinking. This offers practical pathways to improve the online behavior ecosystem and promote mental health development among college students, while also providing more targeted research directions for future studies on the relationship between physical activity and cyberattack behavior.

## Data Availability

The raw data supporting the conclusions of this article will be made available by the authors, without undue reservation.
